# Microalgae as Raw Materials for Aquafeeds: Growth Kinetics and Improvement Strategies of Polyunsaturated Fatty Acids Production

**DOI:** 10.1155/2023/5110281

**Published:** 2023-01-06

**Authors:** Oscar Soto-Sánchez, Pamela Hidalgo, Aixa González, Patricia E. Oliveira, Adrián J. Hernández Arias, Patricio Dantagnan

**Affiliations:** ^1^Departamento de Procesos Industriales, Facultad de Ingeniería, Universidad Católica de Temuco, Temuco, Chile; ^2^Núcleo de Investigación en Bioproductos y Materiales Avanzados, Departamento de Procesos Industriales, Facultad de Ingeniería, Universidad Católica de Temuco, Temuco, Chile; ^3^Núcleo de Investigación en Producción Alimentaria, Departamento de Ciencias Agropecuarias y Acuícolas, Facultad de Recursos Naturales, Universidad Católica de Temuco, Temuco, Chile

## Abstract

Studies have shown that ancient cultures used microalgae as food for centuries. Currently, scientific reports highlight the value of nutritional composition of microalgae and their ability to accumulate polyunsaturated fatty acids at certain operational conditions. These characteristics are gaining increasing interest for the aquaculture industry which is searching for cost-effective replacements for fish meal and oil because these commodities are one of the most significant operational expenses and their dependency has become a bottleneck for their sustainable development of the aquaculture industry. This review is aimed at highlighting the use of microalgae as polyunsaturated fatty acid source in aquaculture feed formulations, despite their scarce production at industrial scale. Moreover, this document includes several approaches to improve microalgae production and to increase the content of polyunsaturated fatty acids with emphasis in the accumulation of DHA, EPA, and ARA. Furthermore, the document compiles several studies which prove microalgae-based aquafeeds for marine and freshwater species. Finally, the study explores the aspects that intervene in production kinetics and improvement strategies with possibilities for upscaling and facing main challenges of using microalgae in the commercial production of aquafeeds.

## 1. Introduction

It is a well-established knowledge that microalgae have been used as raw material to manufacture various products since ancient times. For example, blue microalgae *Spirulina* consumption can be traced well into the past in different parts of the world. According to the chronicles of the Spanish conquistadors, the ancient Aztecs collected *Spirulina* from the surface of Lake Tenochtitlan [1]. In this sense, reviews made by Koyande et al. [2] and Anis et al. [3] indicate that in geographical areas of Asia and America, species of microalgae (e.g. *Gracilaria lichenoides*, *Gelidium corneum, Chlorella,* and *Spirulina* species) were consumed about 2000 years ago as healthy functional foods.

Microalgae may be photoautotrophic (performing photosynthesis), requiring only the presence of light and a source of inorganic carbon (e.g. CO_2_) to reproduce. At the other extreme, they may be heterotrophic, requiring more complex organic compounds as a carbon source and not depending on light for reproduction [4]. Photoautotrophic microalgae are microorganisms containing principally chlorophyll-a, meaning that they are capable of photosynthesis. Most of them are single-celled, and can form chains, colonies, or coenobia; they account for most of the organic material in aquatic ecosystems. They are responsible for almost 50% of the total photosynthesis of the planet [5, 6].

Microalgae and their products have been used for various purposes in different industries, such as cosmetics, wastewater purification, drug production, energy, and a source of nutrients for human and animal foods and feeds [7]. The aquaculture industry has a rising interest in using microalgae (whole cells or their by-products) as a source of nutrients due to their rapid growth and high lipids content. They are of great value, especially in marine aquaculture, for feeding the larval stages of shrimps, fish, and mollusks. There are more than 30,000 species of microalgae, of which barely 100 have been studied, and less than 30 are exploited for commercial purposes [8].

Microalgae can contain a plethora of bioactive metabolites, and thus many of the products obtained from the microalgae culture have a nutraceutical value. In recent years, one focus of particular interest has been the production of polyunsaturated fatty acids (PUFA). The importance of these essential biomolecules lies in their benefits for human and animal health, as they play a structural and functional role in metabolism [9, 10]. Eicosapentaenoic acid (EPA) and docosahexaenoic acid (DHA) are essential constituents of phospholipids, the main components of cell membranes. Long-chain polyunsaturated fatty acids (LC-PUFA) such as EPA and DHA are structural components of cell membranes that provide functional properties which affect physicochemical parameters of the cell and influence membrane proteins and their functions [11, 12].

Likewise, when they form components of triacylglycerols (TAG) and other storage lipids, the LC-PUFAs of the type *ω*-3 act as energy reservoirs, which are then recovered through the *β*-oxidation of fatty acids in the mitochondria [13]. They have other, more specific functions in regulating the metabolism, in natural form or as derivatives. This regulation may be extracellular through eicosanoid compounds like prostaglandins, leukotrienes, resolvins, maresins, and protectins, or intracellular ligands that control gene expression [14, 15]. In marine fish like turbot (*Psetta maxima*) and the larvae of gilt-head bream (*Sparus aurata*), these fatty acids are related with optimum growth, sharp sight, the capacity of the larvae to capture their prey, resistance to diseases, and promoting survival [14, 16–21]. In Atlantic salmon (*Salmo salar*), they moderate gene expression in inflammatory and immune response processes [22]. Eicosanoids appear to play the same physiological roles in fish reproduction as they do in mammals [13].

The World Health Organization has published countless times that cardiovascular diseases are the leading cause of death worldwide. These conditions are thought to be related to low ingestion of *ω*-3 LC-PUFA, principally EPA and DHA [23]. These types of fatty acids have known effects, and it is generally accepted that they protect against a wide range of pathologies–including cardiovascular and inflammatory diseases [24–29]. They also have a recognised role in protecting against other chronic and metabolic diseases, such as diabetes, obesity, osteoporosis, neurological degeneration, and bone fractures [30–34]. They contribute to foetal development [35] and relieve symptoms of depression and anxiety [36, 37]. The United Nations Food and Agriculture Organisation has reported that microalgae have shown potential as an alternative and sustainable source of *ω*-3 LC-PUFA in food for aquatic organisms [38].

PUFA divides into two families, *ω*-3 and *ω*-6, including 18-carbon fatty acids in their chains. LC-PUFA of the *ω*-3 family (EPA, 20:5 *n*-3 and DHA, 22:6 *n*-3) can be biosynthesised from short-chain fatty acids (SC-PUFA) like *α*-linolenic acid (a-ALA, 18:3 *n*-3). In contrast, LC-PUFA of the *ω*-6 family (e.g. ARA, 20:4 *n*-6) is biosynthesised from linoleic acid (LA, 18:2 *n*-6). LC-PUFA is essential for human and animal nutrition. LC-PUFA, especially those of the *ω*-3 series, is a vital nutrient for humans and animals' health, growth, and development [28, 39].

Long-chain polyunsaturated fatty acids are formed by the desaturation and elongation of *α*-linolenic acid [40, 41]. Aquatic–principally marine–animals are unable to synthesise LC-PUFA themselves due to the low activity, or absence, of enzymes responsible for lengthening the chains; for this reason, these acids have to be supplied as a food supplement [42]. On the other hand, freshwater fishes can convert short-chain PUFA into long-chain PUFA, such as are found in EPA and DHA, although this ability varies from one species to another [17]. The biosynthesis of LC-PUFA begins mainly in the endoplasmic reticulum of the hepatic cells when *Δ*6 desaturase adds a double bond in the sixth position of the C-C bond of the -COOH end of linoleic acid and linolenic acid to generate *γ*-linolenic acid (GLA) and stearidonic acid (SDA, C18: 4, *n*-3), respectively. These fatty acids then lengthen to produce dihomo-*γ*-linolenic acid (DGLA, C20: 3, *n*-6) and eicosatetraenoic acid (ETA, C20: 4, *n*-3), respectively, by means of *Δ*6 elongase. Finally, *Δ*5 desaturase adds a double bond in the fifth C-C bond causing further desaturation to produce ARA and EPA. EPA undergoes two successive elongation cycles, generating first docosapentaenoic acid (DPA, C22: 5, *n*-3) and then tetracosapentaenoic acid (C24: 5, *n*-3), which subsequently produces tetracosahexaenoic acid (THA; C24: 6, *n*-3) through *Δ*6 desaturase. This 24-carbon PUFA undergoes *β*-oxidation, shortening its chain by two carbons to produce DHA, the final product [43, 44].

The lipid and fatty acid profiles in organisms depend considerably on the type and quantity of feed consumed, leading to improved fish development and growth; inclusion of these compounds in fish diets helps to diminish the number of skeletal deformations [16, 45], has a positive effect on the development of the digestive tract and enzymatic activity [46], and increases resistance to stress [10, 47].

The subject of this review is to present different investigations into microalgae oriented towards the aquaculture industry, including details of the production kinetics from composition to current improvement strategies for producing polyunsaturated fatty acids, and the response of several species fed with aquafeeds based on microalgae.

## 2. Species of Microalgae Used to Produce Polyunsaturated Fatty Acids

Microalgae have a complex biochemical composition due to the presence of the photosynthesis apparatus, which enables them to produce a great wealth of important bioactive compounds, such as pigments, vitamins, and long-chain polyunsaturated fatty acids [5, 48]. Due to the essential benefits that they offer, research has focused on the search for new alternative sources for the production of *ω*-3 LC-PUFA. Microalgae as primary producers of *ω*-3 PUFA in marine ecosystems have demonstrated the potential to reduce dependence on conventional raw materials for aquaculture diets [45, 49–52]. Many microalgae can accumulate lipids, making up between 20 and 50% of their dry weight [26, 53–55]. Nevertheless, when microalgae are subjected to stress, they can produce up to 85%, depending on the growth conditions and the producer species [56–61]. Furthermore, these microalgae can provide essential vitamins (B6, B12, and folic acid) and natural pigments (*β*-carotene, astaxanthin, and diterpenes) [51, 62].

Various marine microalgae and macroalgae species belonging to the divisions Phaeophyceae, Rhodophyceae, Bacillariophycea, Dinophyceae, or Chlorophyceae, contain high amounts of PUFA. EPA predominates among different microalgae species like Eustigamatophyceae and Prasinophyceae, while DHA is commonly found in some dinoflagellates like *Crypthecodinium cohnii*, as well as *Schizochytrium* and related species [63, 64].

Microalgae can also synthesise large quantities of bioactive compounds like organic acids, carbohydrates, amino acids and peptides, vitamins, growth substances, antibiotics, and enzymes [26, 65]. The preparation of feed ingredients with microalgae was first promoted because of the high protein content, around 50%, found in general like *Chlorella*, *Scenedesmus*, *Arthospira*, etc. According to Tibbetts [66], most found amino acids in microalgal proteins are leucine (3-14 g100 g^−1^ protein), arginine (2-14 g100 g^−1^ protein), and lysine (2-10 g100 g^−1^ protein). Furthermore, protein in microalgae presents a higher nutritional value than in various frequently consumed kinds of cereal and legumes. However, it is not higher than that found in animal sources [67]. The majority of products in the market today are produced mainly from dry biomass microalgae. Although most of the results obtained are promising, there are cases of side effects and allergies which cause intestinal disorders in organisms and reduce digestibility. Some of the reasons are related to the tridimensional structure of the proteins, inadequate processing, and antinutritional components present in the cell [68].

Further efforts are therefore needed to isolate proteins in their native form and determine their bioactivity. One problem is that the sensitivity of proteins extracted from microalgae to the extraction and isolation processes may lead to their denaturation; consequently, most investigations observe the effects of the peptides obtained by hydrolysis induced with specific enzymes obtained from plants, microorganisms, and animals [69]. One study observed that the number and sequence of amino acids are dominant factors; thus, a wide variety of results could be obtained from the possible combinations, grouped into four types: antioxidant, antihypertensive, hypolipidemic, and antimicrobial [70].

Table 1 below presents a summary of the yields reported over the years for some of the main EPA- and DHA-producing microalgae [71].

Another type of PUFA produced by some microalgae is arachidonic acid (ARA). It is produced by heterotrophic species, i.e. those that require organic compounds and grow in the absence of light. Both ARA and DHA are principal components of the phospholipid membrane of the brain; they can act as immunosuppressors and induce inflammatory responses, blood clotting, and cell signalling. ARA deficiency in the organism can cause hair loss, degeneration of the fatty liver, anaemia, and reduced fertility in adult humans. ARA also acts as a natural antifreeze in aquatic Arctic animals and terrestrial animals like reindeer that feed on mosses [10, 72].

*Porphyridium purpureum*, a single-celled heterotrophic red alga, is one of the few microalgae that produce a significant quantity of ARA–which may exceed 30% of its total fatty acids [73]. When this species grows under conditions of stress (less than optimal light intensity, pH and temperature, and increased salinity and scarcity of nutrients), ARA production may be up to 40% of its total fatty acids, while if cultivated under favourable growth conditions, LC-PUFA are primarily represented by EPA [74]. The green freshwater microalga *Parietochloris incisais* is considered one of the richest plant sources of ARA, with 77% of the total dry weight [75]. Red microalgae have been used to test the effects of different environmental and growth conditions on the production of various fatty acids, especially ARA. The ARA production of the algal species *P. purpureum*, *P. cruentum*, *Ceramium rubrum*, and *Rodomella subfusca* has ranged between 40 and 60% of their total fatty acids [44, 76].

## 3. Culture Conditions of Microalgae to Promote PUFA Production

The efficiency in PUFA production from microalgae can be affected by factors such as: culture stress conditions, pH of medium, and nutrient concentration (glucose, nitrogen, or added salts) [60, 63, 77, 78]. These effects have improved industrial production of microalgae enriched with PUFA, such as the use of nondrinking water for cultivation [79]; cultivation independent of the seasons [80]; and flexibility and adaptability to changes in culture conditions [81].

### 3.1. Temperature

Culture temperature is one of the main factors affecting microalgae growth and lipid accumulation. Temperature influences all metabolic processes [82–84]. The photosynthesis, respiration, and growth rates of microalgae diminish when optimum temperatures are exceeded, due to imbalances between adenosine triphosphate (ATP) production and energy demand, inactivation or denaturation of proteins needed for photosynthesis, or stress in photosystem II activity. Temperature also alters the composition of fatty acids [85]. The temperature used may range from 15 to 28°C, and the pH from 7 to 8, depending on the microalgae species. In this temperature range, it is observed that the culture conditions that encourage PUFA production are light irradiance between 50 and 300 *μ*mol photons m^−2^s^−1^, air enriched with CO_2_ 0-1% (v/v), and restricting nitrogen and phosphorus [86].

Temperature control is a challenge for the industrial production of microalgae in open-air photobioreactors. Using strains with tolerance to high temperatures can reduce production costs as active temperature control is not required. Barten et al. [87] isolated photoautotrophic marine microorganisms to reduce the need to control high temperatures. They worked with 22 samples collected around the Caribbean island of Bonaire. They used a temperature of 40°C to apply selective pressure during strain enrichment, and the strains with the highest growth rates were selected. They identified 59 strains, of which they selected five to characterise their growth rate and biomass composition. *Picochlorum sp*. and *Leptolyngbya sp*. presented optimum growth at 40°C and 35°C, respectively, with a growth rate of 0.12 h^−1^ during the day. The strains showed 62.1% and 68.2% of protein and contained various fatty acid compositions suitable for application as edible oil and biofuel.

In other investigations, the unsaturated fatty acid contents diminished with increasing temperature. It is reported that the species *Nannochloropsis salina* produces higher contents of PUFA, such as EPA, under suboptimum temperature conditions (15°C) [88]. Unsaturated fatty acids are necessary for microalgae to maintain the membrane's fluidity, which in turn depends on the length of the fatty acid chains [85].

Ribeiro et al. [89] presented a mathematical and computer model to calculate the temperature profiles in a transitory state of a prototype compact tube photobioreactor to increase the fatty acid production from microalgae. The model combined theoretical concepts of classical thermodynamics with theoretical and empirical correlations of fluid mechanics and heat transfer. The physical domain is the volume element model through which the physical system (tubes of the photobioreactor) is grouped such that the ordinary differential equations depend solely on time. The energy interactions between the volume elements are established by empirical correlations of heat transfer by conduction, convection, and radiation. The proposed mathematical model considers the variations in temperature between the volume elements of the wall of the tube photobioreactor caused by the flow of liquids, solar radiation, and environmental interactions. For each VE_*w*_ (volume element in the tube walls), the energy balance in the wall of the plastic tubes was calculated by the equation:

(1) $$\begin{array}{c} {\dot{Q}}_{\text{rad}}^{\left(j\right)}-{\dot{Q}}^{\left(j\right)}-{\dot{Q}}_{\text{air}}^{\left(j\right)}=m_{w}^{\left(j\right)}C_{w}\frac{dT_{w}^{\left(j\right)}}{dt}, \end{array}$$

where *C*_*w*_ is the specific heat of the plastic wall (Jkg^−1^K^−1^), *m*_*w*_ is the mass of the wall (kg), and ${\dot{Q}}_{\text{rad}}$ is the solar radiation that reaches the walls of the photobioreactor tubes (Wm^−2^). The following equation calculates the heat transfer between the wall and the air ${\dot{Q}}_{\text{air}}$):

(2) $$\begin{array}{c} {\dot{Q}}_{\text{air}}^{\left(j\right)}=h_{e}A_{e}\left(T_{w}^{\left(j\right)}-T_{\infty }\right), \end{array}$$

where *h*_*e*_ is the coefficient of heat transfer by convection between the exterior wall and the ambient air (Wm^−2^K^−1^), *A*_*e*_ the area of the exterior wall (m^2^) and *T*_∞_ is the air temperature (K).

Predicting the temperature profiles along the length of the tubes and their position with respect to the light source (sunlight) is of great importance for the design of bioreactors of this type. The volume element method was used to formulate the problem and solve the resulting ordinary differential system of equations with a Runge-Kutta fourth-order method. The results showed that the current model and the numerical instrument could predict the incident radiation and temperature variations inside the reactor (between 19 and 23°C); therefore, it is expected to be a valuable instrument for the simulation and design of photobioreactors to process microalgae.

### 3.2. Nutrients

To grow, microalgae need water, light, CO_2_, and mineral salts. The latter must include a nitrogen source, such as nitrate or ammonium, and a phosphorus source, usually an inorganic phosphate. According to Santin et al. [10], culture media composition and growth phase can affect lipid metabolism. The nutrients have a direct impact on the composition and productivity of the PUFA produced by the microalgae [83, 90, 91]. Phosphorus is an essential macronutrient, playing a role in growth, metabolism, and other vital processes for microalgae such as photosynthesis, respiration, ATP signalling, and synthesis [92]. Liang et al. [93] reported that the species *Scenedesmus abundans* grew in all the different phosphate concentrations (20, 40, 60, and 80 mgL^−1^) tested in the culture medium. The lag phase of culturing lasted three days and was followed by the logarithmic phase; the stationary phase was reached on the fifteenth day of the growth period.

The productivity of fatty acids from microalgae is associated with both the lipid content and the biomass production. However, the accumulation of lipids needs a limiting nitrogen level in a solution medium, while good growth of microalgae requires sufficient nitrogen, and both need adequate carbon nutrients. High lipid productivity can only be achieved by determining a suitable C:N ratio in a solution medium. Lipid productivity of *Chlorella vulgaris* in mixotrophic culture was studied by contrasting glucose, phosphorus, and nitrogen concentrations. Phosphorus has a strong influence on the growth of *C. vulgaris* but has little impact on lipids accumulation. Simultaneous deficiency of nitrogen and sufficiency of organic carbon can promote the accumulation of lipids in algae. A high lipids content (23.9%) was achieved in a culture with limited nitrogen (0.1 gL^−1^ NaNO_3_) and a sufficient supply of glucose (10 gL^−1^ glucose). The culture's initial C:N ratio played a critical role in the microalgae accumulation of lipids [94].

A phosphorus limitation stimulates the activity of *Δ*6-desaturase and promotes the conversion from palmitic acid to LA (18:2 *n*-6). In addition, stimulation of the *ω*-6 pathway promotes the biosynthesis of ARA causing a detriment of the EPA pool [10, 74, 95].

The Monod kinetic model is most frequently used to describe microalgae growth under low and moderate concentrations of nutrients; under high concentrations, it can only explain growth inhibition. To overcome this limitation, a modification was introduced into the Monod model based on the Haldane model of enzyme inhibition [96]. The Monod model is generally applied in studies of the specific growth rate in microalgae as a function of one of the three principal substrates: inorganic carbon, nitrogen, or phosphorus [97–99]. The model does not consider growth limitation by multiple nutrients. However, existing studies have shown that two or more substrates could limit microbial growth rates, and this finding could be extended to explain the limitation of microalgal growth by multiple nutrients [100]. In particular, microalgae growth is inhibited when the NH_3_ concentration is higher than 300 mgL^−1^ in the culture solution [96].

The Luedeking-Piret model for product formation kinetics fits the production of neutral lipids in microalgae [101–104]. He et al. [101] described the culture of the microalga *Isochrysis galbana* in a flat plate photobioreactor and reported that the Luedeking-Piret equation can describe its growth. The coefficient associated with growth (*α*) increases with biomass production, suggesting the formation of lipids by *I*. *galbana.*

### 3.3. pH

One of the determining parameters for microalgal cultivation is the pH, which establishes the solubility and availability of CO_2_ and nutrients. During cultivation of microalgae, pH rises due to uptake of inorganic carbon, which can cause cell growth inhibition at a certain value of pH-dependent of the microalgae species [105, 106].

Both biomass productivity and accumulation of lipids depend on pH [105, 107, 108]. An interesting study reported by Abinandan et al. [109], evaluated the toleranz of *Desmodesmus sp*. (MAS1) and *Heterochlorella sp*. (MAS3) under different Cd concentrations. The results revealed a better microalgae growth response between 1 and 5 mgL^−1^ of Cd than for 10 and 20 mgL^−1^ at a low pH (3.5). In addition, the authors detected an increase of lipid accumulation (around 10%) in comparison to the control (without Cd), obtaining a biomass enriched with lipids. However, Shah et al. [108] have observed a rise in cell density at pH between 7 and 9 for *Pavlova lutheri.* Another key point is that a significant accumulation of lipids (32-35%) occurred also at the same pH range, corresponding almost a third to unsaturated fatty acids.

Several authors have used bicarbonate (HCO_3_^−^) as an inorganic carbon source for microalgae to control pH and reduce the loss of CO_2_ [106, 110–112]. In this context, Roa et al. [111] studied a batch culture system for *Chlorella sp* in the presence of HCO_3_^−^. They aimed to evaluate the growth kinetic of this microalga in an alkaline medium (pH = 8.1), constant light, and stirring. The authors obtained unexpected results, reporting that *Chlorella sp*. reduced the growth rate by over 50% with the addition 0.29 M of HCO_3_^−^. Based on this observation, it is assumed that the addition of bicarbonate limited microalgae growth and further studies were recommended to evaluate lower concentrations. In a similar manner, Song et al. [112] also observed not only that the presence of bicarbonate affects the *Chlorella* L38 growth but also that the alkaline values of culture media (9.5-10) provoke an increase of ammonia release. Therefore, they proceeded to control pH (7-8), achieving an increase of biomass production of 67% and a release rate of ammonia lower than conventional absorption processes (43.1%). Noteworthy, the authors in this study used a smart system (called hybrid absorption-bioconversion) for the production of bicarbonate. The solution of bicarbonate came from an absorption column used for capturing CO_2_ from flue gas with ammonia, obtaining NH_4_HCO_3_. This system proved experimentally to be a cost effective process.

### 3.4. Irradiance

The effect of light intensity on lipid content, growth, and fatty acids composition has been studied in various microalgae species [10, 113–115]. Increasing the light intensity may either increase or diminish the total lipids content. Some reports show an increase in total lipids, particularly DHA, with greater light intensity [116]. In contrast, other reports indicate that microalgae perform photosynthesis very efficiently in low light conditions. However, Santin et al. [10] state that saturated fatty acids tend to increase at high light irradiance, whereas PUFAs concentration tends to decrease or remain stable. They cannot achieve efficient photosynthesis under intense sunlight because the cells absorb more light energy than they can convert into biochemical energy dissipating part of this energy in the form of heat [117].

Oxygen is a crucial environmental factor for the synthesis of *ω*-3 LC-PUFA in microalgae. In general, a high oxygen supply is beneficial for cell growth; however, it obstructs the synthesis of PUFA. The generation of reactive oxygen species in aerobic conditions leads to the peroxidation of lipids, especially in PUFA. Lipid profile of many species is altered under oxidative stress [118].

The photoperiod affects the growth of cultures and the accumulation of lipids in microalgae. It has been observed that the maximum specific growth rate (*μ*max) achieved under 24 h illumination is 0.12 day^−1^, with a lipid content of 35%, compared with 0.1 day^−1^ and a lipids content of 15% under dark growth conditions [108].

Other researchers have used blue and red light-emitting diodes (LED) to study the effects of mixing different wavelengths proportions, varying photoperiod regimes and stressing cell biomass with green wavelength on the cell biomass and lipid production of species such as *N. salina*, *I. galbana*, and *Phaeodactylum tricornutum*. The maximum specific growth rates of *I. galbana* and *P. tricornutum* were obtained with a mixture of 50:50 blue and red LED light; in *N. salina*, the maximum was achieved under red LED light. The maximum cell biomass from *N. salina* was 0.75 g dcwL^−1^, and for *P. tricornutum,* 1.07 g dcwL^−1^, obtained with a light:dark cycle of 24:0 h. However, the maximum biomass from *I. galbana* was 0.89 g dcwL^−1^ in a light:dark cycle of 18:6 h. After exposure to green LED, the maximum lipid contents for *N. salina*, *I. galbana*, and *P. tricornutum* were 49.4, 63.3, and 62.0% (w/w), respectively. EPA and DHA were obtained at 1% in *P. tricornutum* and 2% in *I. galbana* [119].

### 3.5. Carbon: Nitrogen Ratio

Huang et al. [120] carried out a study to improve the proportion of carbon to nitrogen (C:N) in biomass from microalgae collected to produce better biogas by fermentation. Biodegradable cation starch with a high C:N was synthesised to harvest *C. vulgaris*. The authors also studied the pH impact since the zeta potential of both the microalgae and the cationic starch would change with the pH. The results indicated that cationic starch could be used to harvest more than 99% of the microalgae, and the C:N ratio could be increased from 7.50 to 7.90. The zeta potential of the microalgae always remained negative, and presented a tendency to fall at first and then improve. The maximum amount of microalgal biomass that could be flocculated by 1 g of cationic starch was 8.62 g, with the aid of autoflocculation at pH 3. The concentration of flakes formed at pH 11 was 25.74 gL^−1^ and their diameter was 0.553 mm, much larger than the flakes formed at pH 3 (0.208 mm).

Khanra et al. [121] evaluated the influence of the C:N ratio on the mixotrophic growth of microalgae and the role of nanomaterials in cell recovery and improving lipid content. In this study, three species of microalgae, selected from local freshwater bodies, were isolated and identified as *Chlorococcum sp.*, *Scenedesmus sp*., and *Euglena sp*. The three species were evaluated for lipid assimilation by means of the autotrophic and mixotrophic growth, achieving a better performance *Chlorococcum sp.* for mixotrophic growth with nitrate. The authors also proceeded to evaluate various organic carbon sources. Sucrose proved to be the best source for improving microalgal biomass production (3.5 gL^−1^) and lipid content (58.35%). Subsequently, response surface methodology-Central composite design was used to optimise the C:N ratio. Experimental results achieved a maximum biomass production of 5.02 gL^−1^, as well as an increased lipid content of 60.34% at C:N around 1.7. The study also analysed the addition of Ti nanoparticles (Ti nps) to the microalgae culture to improve the lipid content and the sedimentation of the biomass by flocculation. The best concentration, 15 ppm of Ti nps, resulted in a flocculation efficiency of 82.46% at a sedimentation time of 45 min and an increase of lipid content (74.29%), corresponding 29.06% to unsaturated fatty acids.

## 4. Kinetic Models of Microbial Growth Applied to Microalgae

Today, the field of predictive microbiology is attracting great interest. It is used to describe, predict, evaluate, optimise, and develop biological processes without the need to pass through the experimental stage. It requires correlations and parameters associated with processes; kinetic growth models play a fundamental role in this type of research [122]. Many different models have been proposed; however, selecting an appropriate model for simulating microalgae growth is challenging [123, 124]. The following kinetic bacterial growth models are some of the most important that apply to microalgae growth process in bioreactors:

### 4.1. Monod Equation

This model, developed in 1942, describes a ratio between microbial growth and a substrate that limits growth but without inhibiting it in any way during the growth process. The model only considers the positive phases of growth, and the microbial population is assumed to be homogeneous [125]:

(3) $$\begin{array}{c} \frac{dX}{dt}={X\mu }_{\text{max}}\frac{S}{K_{s}+S}, \end{array}$$

where *μ* is the specific growth rate of the microorganisms; *μ*_max_ is the maximum specific growth rate of the microorganisms; *S* is the concentration of the growth-limiting substrate; *K*_*s*_ is the “mean velocity constant” (the value of *S* when *μ/μ*max = 0.5 *μ*_max_ and *K*_*s*_ are empirical coefficients of the Monod equation); they differ between species and as a function of environmental conditions.

To emphasise by means of an example, Liu et al. [126] applied the Monod equation to test several nitrogen concentrations and sources on the growth of three algae. The results indicated that all species grew well in medium with nitrate substrate. The values of *μ*_max_ and *K*_S_ for nitrate were 0.71 divisions d^−1^ and 53.55 *μ*mol L^−1^ for *Skeletonema costatum*, 0.67 divisions d^−1^ and 23. *μ*mol L^−1^ for *Prorocentrum micans*, and 0.23 divisions d^−1^ and 17.57 *μ*mol L^−1^ for *Chattonella marina*.

### 4.2. Moser Model

This model originated as a modification of the Monod model in 1958; it is considered a good approach when the cell composition is independent of the processing time. Its formulation is implicit in consideration of the effect of the propagation of mutant species in the bacterial population. This model is represented by the following equation:

(4) $$\begin{array}{c} \mu =\mu_{\text{m}\overset{´}{\text{a}}\text{x}}\frac{S^{n}}{K_{s}+S^{n}}. \end{array}$$

It considers the same variables as the Monod model, and the introduction of *n*, an empirical parameter. The *n* value is dependent on every process, but indicates for *n* = 0, that the cell growth occurs without inhibition, while for *n* ≠ 0, there is some kind of inhibition.

In this context, Sharma et al. [127] presented a study that optimises stirring speed, aeration time and substrate concentration (pretreated *Rhizoclonium sp*. algal hydrolysate**)** for the production of ethanol using *Saccharomyces cerevisiae*. First, the results showed that the Moser model provided a precise pathway for mathematical modelling of ethanol production experimental data. Second, the operational conditions of 200 rpm, aeration time 8 h, and substrate concentration of 40 gL^−1^ resulted in an optimal ethanol production of 37.34 gL^−1^.

### 4.3. Contois Model

Developed in 1959, this model has been widely used in the literature to describe anaerobic processes, mainly for cases in which the hydrolysis stage exerts greater control over fermentation speed. It also considers that the specific growth rate depends on the population density in the medium:

(5) $$\begin{array}{c} \mu =\mu_{\text{m}\overset{´}{\text{a}}\text{x}}\frac{S}{K_{s}X+S}. \end{array}$$

Like the models described above, this model depends on a balance of biomass and substrate; where *X* represents the concentration of biomass, *S* represents the carbon source concentration, and *μ*_max_ is the maximum specific growth rate of the microorganisms.

The article published by Yunardi et al. [128] shows the application of an algae growth model for the cultivation of *Chlorella kessleri*. The postulated mathematical growth model proposed for *C. kessleri* was developed from the already validated growth study of Contois, and this was modified to include a second substrate as a factor that influences the development of cell biomass. The resulting differential equations and the modification of the Contois model were solved numerically with the Runge-Kutta method. The results of the study demonstrated the validation of the original Contois model with predictions of the concentrations of cells and substrates, however, some values obtained deviate from the experimental data. With the modification of the Contois model considering the second substrate for the cell growth of *C. kessleri*, in addition to nitrate as the first substrate, the predictions yielded an excellent result with the experimental data.

Other models such as the logistic and modified logistic models have been proposed. Huang et al. [129] fitted experimental data with increasing substrate concentration. They obtained that logistic and modified logistic equations predict better biomass growth. However, a modified logistic equation in combination with a modification of the Monod equation could better describe the relationship between the specific growth rates and the substrate concentrations. For more information about these equations references we encourage the readers to check the cites Shuler and Kargi [130], Weisstein [131] and Kargi [132].

## 5. Production systems

An interesting study by Hamilton et al. [133] showed that aquaculture generally supplies 800,000 tons of EPA/DHA per year for human consumption. This figure is less than the human nutritional demand that reaches just over 1 million tons that are required to supply the world population with 0.5 g of EPA + DHA per day. This low value is more worrisome when considering the exponential growth of the world population.

World fish populations are known to be diminishing; as a result, fish captures for human consumption might fall in the future. Furthermore, some marine fish species, like salmon, sardines, tuna, anchovy, mackerel, and hake, are sometimes contaminated with heavy metals like copper or mercury, organic pollutants like PCB (polychlorinated biphenyls), and dioxins, which have toxic effect on human health. For this reason, algae culture is considered an alternative source for the production of *ω*-3 LC-PUFA [5].

Large-scale culturing of microalgae would contribute decisively to developing a sustainable PUFA production industry, generating profitable, high-value products. There are a large number of microspecies that have the potential for large-scale cultivation, but there are not enough applied studies to conduct commercial trials. It is necessary to estimate large amounts of microalgae to compete with other raw materials for the sustainable production of polyunsaturated fatty acids. Successful technologies for microalgae culture will need to create large amounts of biomass, making the use of foods for fatty acid production more attractive [134].

For microalgal biotechnology to be sustainable, feasible, and economically viable, successful culture technologies need to be developed for selective biomass production. Viable culturing means that biomass production must be higher than 30 gm^−2^ day [135]. The objective of almost all researchers and companies has been to improve biomass or oil productivity from microalgae. Experimentation has been a good way of studying different technological options. But experimentation demands much time and much money.

The process of cultivating microalgae in artificial reactors is a method that has proven successful for large-scale production. These reactors can be classified as open tanks with aerobic processes and those commonly known as closed photobioreactors. Photobioreactors have a higher s/v ratio. The system considers a larger surface in contact with the light source, reducing the effects of shade and thus offers better control of growing conditions, monitoring the parameters of mass flow, contamination, temperature, pH, gas transfer, and distribution of nutrients. The advantages of aerobic tanks over closed photobioreactors include relatively low cost, easy cleaning, use of nonagricultural land, low energy consumption, and ease of maintenance [136].

In spite of its biomass production, capacity is not very efficient, demanding a large area of land, and is limited to a few strains of algae and easy contamination of crops. Traditional aerobic growing systems also entail higher costs due to slow and slow growth rates and the constant need for aeration as well as light. Aerobic tanks are used in experiments with fewer species of microalgae and have the ability to withstand unfavorable environmental conditions, such as *Chlorella* and *Spirulina*.

### 5.1. Closed and Open Systems

Various production systems have been explored to achieve the production of large volumes of high-quality PUFA. In general, PUFA production strategies have incorporated microalga culture systems as part of production plans, for example, for a specific service [137]. The implementation of large-scale production systems has varied, being limited to microalga products of strong commercial interest [138]. Certain strains of microalgae present different requirements in terms of nutrient intake and supply, as well as in their environmental and culture conditions [53]. Nevertheless, it is recognised that investigations are in the initial stages, and there is still a long way to go [139]. In this respect, efforts have focused on using strains offering a high lipid yield and rapid growth, optimising culture conditions, and using nutrient-rich waste to increase the yields of lipids and biomass [53].

Microalgae are cultured predominantly in photoautotrophic conditions. Culture methods include open methods like open tanks and closed methods like photobioreactors. The choice between them depends strongly on the microalgae strain used [53, 55]. The majority of microalga production systems use culturing in suspension, resulting in diluted suspensions that increase the costs of harvesting and water removal [140].

Of the methods used for PUFA production, open and semiopen systems have been noted to offer high productive potential. For example, comparisons have been made between the performance of microalga production systems based on their lipids productivity. The results establish that chemostats and fed-batch reactors produce similar quantities of lipids [141]. Likewise, some experiments using continuously stirred tank reactors (CSTR) for PUFA production have given good results [142].

Some kinetic conditions may be favourable for closed PUFA production systems. Large-scale production systems depend critically on kinetic parameters; they are designed with these in mind, although PUFA extraction and purification processes are also affected [143].

## 6. Improvement Strategies

Various methodological strategies have been proposed to improve the productivity and composition of PUFAs produced from microalgae. These strategies include taking advantage of the interrelationship dynamics between different species, altering the culture conditions, or the use of appropriate mathematical models to represent the different metabolic mechanisms of production.

### 6.1. Cocultivation

Other works have explored the effectiveness of mixed culture systems for essential fatty acid production. This strategy consists of culturing various microalgae species in the same reactor to establish synergistic coexistence between species. This method has been widely explored, and in general, a more significant accumulation of PUFA is reported than in monoculture systems [144].

Increasing the concentrations of CO_2_ during aeration of mixed cultures has also been investigated and found to improve the proportion of the lipids content in the biomass [145]. Some studies have suggested that PUFA-generating microalgae would also serve for bioremediation, taking advantage of their excellent performance in capturing CO_2_ from the atmosphere [107, 146, 147].

Some critical parameters are included in the growth model, such as light distribution, pigmentation kinetics, nitrogen absorption, and the growth and respiration rates; these may be temperature- or depth-dependent. In continuous culture, the dilution rate and inorganic nitrogen concentration delivered to the system have shown significant effects on microalgal growth [148].

In continuous culture studied by Yuan et al. [148], the dilution rate and the concentration of inorganic nitrogen investigated with the experiment have shown significant effects on the growth of microalgae. Directing the study only in quantitative results, the results of the concentration of total particulate carbon and the concentration of particulate carbon per day were tracked, using 3D figures to show the variable trends. The influence of several typical temperatures (13, 16, 20, and 23°C) was modelled and calculated, observing that biomass productivity increased as the dilution rate (*D*) approached 0.

### 6.2. Stress Conditions

In general, culture conditions are a primary focus for understanding the dynamics of PUFA production in microalgae cultures. Here, exploring anomalous culture conditions was an opportunity to find new situations for improving production efficiency. It has been observed that applying stress conditions such as high salinity, *P* and *N* limitation, and low temperature to a microalga culture favours lipids production, and therefore PUFA production. In this respect, some authors state that manipulating the nutrient concentration is a practical, low-cost method of increasing the intracellular accumulation of lipids and triglycerides [10, 78, 149].

Stress conditions are achieved either by specific effects on particular species or by manipulating the environmental culture conditions at certain levels, increasing PUFA productivity. The effects of chemical species have been explored for chemicals with different modes of action to obtain positive results in PUFA production, including exposure to certain nutritional levels (lack or excess of nutrients) or those with collateral effects on the culture. For example, exposure of microalgae to high salinity positively affected PUFA production [150]; and exposure to mutagens traces such as ethyl methane sulphonate resulted in cultures with remarkable lipids production [151].

Stressing microalga cultures with nitrogen scarcity has also been reported to produce a positive trend in PUFA production, showing significant potential in species with a high lipid content [149, 152, 153]. Authors like Breuer et al. [154] and Bona et al. [155] explored the effects of eliminating nitrogen from the nutrients fed to microalgae, observing that this does not produce an increase in lipids production. Still, it does modify the lipids profile, favouring the formation of monounsaturated fatty acids. Nevertheless, it must be noted that the majority of the studies has been carried out with species of microalgae with significant commercial potential. Other researchers have shown that synergy in reducing the nitrogen and phosphorus contents triggered an increase of up to 3.5-fold of the lipids contents in PUFA-generating microalgae [149, 152]. Positive results have also been reported for PUFA production in conditions of reduced phosphorus and other micronutrients [156].

In environmental conditioners, variations in various operative parameters have been explored, such as photoperiod, stirring, aeration, thermal exposure, and electromagnetic field. These works have shown that the most critical factors for increasing lipids production are aeration velocity, light intensity, and photoperiod. At the same time, one of the most favourable conditions for PUFA accumulation is the low temperature [157]. The application of different electromagnetic fields is a method that has been increasingly used in recent years, and the reported results indicate improved PUFA production [158].

### 6.3. Upscaling

Upscaling PUFA production systems based on culturing microalgae is an attractive objective due to the high value of these metabolites [159]. Laboratory studies of kinetic parameters have been fundamental for this purpose [160].

At an industrial level, large-scale systems offer a great advantage. Efficient mathematical models have been established to base the design of modular photobioreactor systems with continuous serial production [161]. Although progress has been made in upscaling microalga production systems, there are several challenges to optimum modelling of highly complex biological processes [162]. Industrial processes, from production reactors to the various processes involved, depend critically on the metabolic conditions of PUFA accumulation, and it is fundamental to determine these at a laboratory scale [163].

To achieve sustainable production of PUFA-generating microalgae, two conditions need to be developed: first, the design of highly efficient systems that will maximise the sunlight used by the cells while consuming a minimum amount of energy in microalga production and harvesting/processing; second, the use of low-impact materials and nutrients, minimising the use of metal or concrete in the construction of the reactors, and using pure CO_2_ or fertilisers as nutrient sources [164].

In any microalga production system, the heart of the process is the photobioreactor in which the biomass is produced. This must be optimised to intercept the highest quantity of light and allow the cells to use it as efficiently as possible. For this to occur, the culture conditions must be optimised to those required by the particular strain being produced to maximise PUFA generation. The principal culture conditions that need to be controlled include temperature, pH, dissolved O_2_, and nutrient availability [107, 165]. Mixing is necessary to minimise gradients in properties like temperature, pH, and nutrient availability, but mainly to maximise cell exposure to light. To avoid gradients in properties, all that is needed is to ensure mixing times in the range of minutes; while to achieve maximum exposure to light, the mixing times or the frequency with which the cells are exposed to light are in the range of milliseconds. The first can be achieved using reasonable amounts of energy, while the second is impossible without investing enormous quantities of energy [166].

Van der Voort et al. [167] stated that DHA derived from the heterotrophic culture of *C. cohnii* was three to five times more expensive than DHA obtained from fish oil. Expressed in economic figures, the conventional and improved costs of heterotrophic production would be 1.51 and 1.22 USD/kg, respectively. In this sense, heterotrophic production appears to be a more viable and economical option than phototrophic production of microalgae in a tube photobioreactor [168]. The estimated production costs of heterotrophic and phototrophic biomass are 3.80 USD/kg and 5.52 USD/kg, respectively. If biomass from heterotrophic microalgae with high oil content is used to produce EPA/DHA, the difference in the cost/kg of EPA/DHA, in favour of heterotrophic culturing, is wider. Chauton et al. [169] reduced the costs of EPA/DHA production by using a flat plate photobioreactor.

## 7. Use of Microalgae in Aquafeeds

The aquaculture industry is growing at a high rate, even faster than the terrestrial animal production industry [170]. Between 2010 and 2019, global aquaculture related to fish and crustaceans increased by 48%, with revenues of around 259 billion USD [171]. As a result of this increase, ingredient demand for feed formulations has also been raised, compromising the profitability and sustainability of the aquaculture industry.

### 7.1. Main Ingredients of Formulations

Feed is one of aquaculture's most significant operational expenses, as in any other intensive animal production system. Feed efficiency and cost determine the profitability and sustainability of this agribusiness activity. Aquaculture's contribution to global aquatic animal production reached a record 49.2% in 2020 [172]. Fed aquatic animal aquaculture continues to exceed that of nonfed aquatic animals. The percentage contribution by nonfed species declined from more than 50% in 1980 to 27, 8% in 2020, indicating the significance of the formulated feed in the global aquaculture industry and the increasing demand for ingredients [172].

According to Catacutan [173], differences in the environmental and physiological factors as well as in the availability of feed ingredients make any feed formula unique for an aquaculture species. Regardless of the aforementioned factors, final formulation must have no negative effect on growth and survival of species and also in feed cost. Furthermore, excessive amounts of nutrients can be detrimental for the environment and species health, and are expensive for the aquaculture industry. Based on the last comment, Tacon [174] published a manual in which listed the recommended maximum inclusion levels of major feed ingredients in a practical diet for some species.

Fish meal and fish oil are commonly used in aquaculture feed formulations due to their high protein content and balanced amino acids profile, and to high amounts of PUFAs, respectively. In this context, the demand from this animal production sector during 2018 was 73% for fish meal and 71% for fish oil of the global market [66]. The high dependence on these commodities has become a bottleneck for the sustainable development of aquaculture. It has motivated, in part, the search for new sustainable, nutritionally adequate and cost-effective ingredients for aquafeed formulations. With the attention to the Agenda 2030, emerged in 2021 the term “Blue transformation”, which additionally, points out the need for a sustainable, efficient, and equitable aquaculture [172]. Consequently, aquaculture transformation requires finding substitutes for ingredients that meet last restrictions. Therefore, several studies have been focused on completely or partially replacing fish meal and oil for plant protein-based and insect protein-based substitutes [175–177].  Table 2 presented a comparison between some non- and conventional ingredients for potential formulations.

As observed in Table 2, microalgae-based products have a great potential as feed ingredients for sustainable aquaculture. However, Tibbetts [66] emphasizes that proximate composition of microalgae is variable and within a wide range. This variability is associated with their vast biological diversity and also depends mainly on cultivation methods and strategies, harvest and downstream processing. As a result, the aquaculture industry could adopt microalgae-based ingredients for routine use, when last disadvantages are overcome, contributing to a reliable supply, consistent nutrient profile, high nutrient quality, and cost-effective production [66].

### 7.2. Species Response to Microalgae-Based Aquafeeds.

Currently, most commercial formulations for fish species in the aquaculture sector contain less than 50% of fish meal and oil and almost 60% of alternative proteins and grains, and alternative oils [66]. In order to be considered a more environmentally-friendly industry (vs. wild fishing), main efforts of the aquaculture sector are focused on the substitution not only of fish meal and oil but also of proteins derived from terrestrial plants. This behavior is a consequence of the environmental problems associated with the increasing pressure put on fish stocks and fertile soils, contributing in case of terrestrial plants to deforestation and eutrophication. As a result, the research of microalgae is also an alternative to replace terrestrial crops or fish meal is rising [7, 178, 179]. However, results of experimental tests derived from total or partial substitution of fish oil or fish meal have identified some drawbacks such as poor digestibility, antinutritional factors, and heavy metals [7, 66]. In contrast, Nagappan et al. [178] has reported that microalgae-based feeds could contribute to better nutritional fish fillets.

In Table 3, the response of different aquaculture species fed with microalgae-based formulations. It is observed that some microalgae are included in the formulation to replace fish meal; nevertheless, results indicated that lipidic profile is improved as a positive consequence.

From Table 3, it is observed that *Schizochytrium sp.* is mainly used to replace fish oil because of their high content in DHA. In fact, Tibbetts [66] also agreed with the last commentary. In addition, this author mentioned other microalgae that could be of great concern for potential aquaculture formulations such as *Crypthecodinium, Isochrysis*, *Nannochloropsis*, *Odentella*, *Tetraselmis*, and *Ulkenia*.

The inclusion of microalgae could significantly increase the content of DHA, EPA, or both. However, several authors stated that this increase should be controlled because an imbalance between DHA and EPA could destroy the balance and structure of the cell membrane of fish larvae. Under this circumstance, the growth performance and quality of the fish larvae are affected [180]. Another interesting point is that processing microalgae by means of extrusion favors the digestibility of dry matter, protein, and energy supplied by microalgae [181] and consequently survival, weight gain, and growth parameters are not compromised.

Main studies shown in Table 3 reported acceptable results to replace fish oil by microalgae with inclusions in aquafeed formulations between 2.4 and 20%. A recent meta-analysis made by Ling et al. [182] focusing on spirulina meal supplementation in fish and shrimp showed significant increase of body weight, specific growth rate, and protein efficiency. In addition, the authors obtained that optimal inclusion levels of spirulina meal should be in the range of 1.46 and 2.26% for fish diets, whereas for shrimp diets up to 1.67%. Based on last comment, research on this subject and homogeneous quality of the large-scale microalgae production and their costs is still needed to ensure a continued growth of the aquaculture industry.

There is a fact that obtaining food products derived from microalgae cultivation could be a good economic alternative, not only as a quality nutritional replacement for fishmeal and fish oil, but also as protection of sustainability standards in aquaculture [170]. In general, reducing the cost of microalgae cultivation can improve the market competitiveness of aquaculture hatcheries [183]. For example, Jannel et al. [184] says that the main limitation of the use of microalgae in aquaculture and animal feed is the high cost of commercial products, which is currently estimated at 7,000 USD/kg, which, with an appropriate geographical location, could be reduced to 1,800 USD/kg.

The cost comparison between conventional feeds and microalgae-based aquafeeds is complex, since the use of microalgae is not as widespread at an industrial level. However, cost estimates have been made comparing feeds formulated based on microalgae biomass with commercial feeds, observing a decrease of 26% in the former compared to the latter. This would significantly affect the profit margin of fish farmers [185]. Therefore, the cost analysis is still under development, as several production, postprocessing, and storage variables need to be further investigated [186].

## 8. Conclusion

This review has given an account of the significance to include microalgae in aquaculture feed formulations. The analyses of main studies agreed that microalgae have adequate nutritional composition for this purpose. The present study has shown the ability of microalgae to accumulate polyunsaturated fatty acids under certain operational or stress conditions, being the most interesting fatty acids related to *ω*-3 and *ω*-6 families such as EPA, DHA, and ARA.

This detailed literature review has compiled current investigations dealing with the production kinetics from composition to current improvement strategies for increasing polyunsaturated fatty acids content in microalgae. However, this research concludes that despite the advancement of science and its achievements in the experimental scale regarding microalgae, to accomplish the requirements of the aquaculture industry, a huge step is needed for large-scale production of microalgae with high content of polyunsaturated fatty acid. In this context, several authors suggest that research should be focused on reliable supply, consistent nutrient profile, high nutrient quality, and cost-effective production of microalgae to be considered as a feed substitute in the aquaculture sector. Experimental tests of great importance have shown that poor digestibility could be improved by the extrusion process of microalgae.

Altogether, issues such as the high availability of biomass from microalgae, their nutritional composition—especially polyunsaturated fatty acids-, and a potential cost-effective production would allow the adoption of microalgae in the aquaculture sector. By this means, the aquaculture industry will—in a sustainable way—meet current and future demands of the global population.

## Figures and Tables

**Table 1:** Content of EPA and DHA polyunsaturated fatty acids in microalgae.

Microalga	EPA 20:5 (%) (eicosapentaenoic acid)	DHA 22:6 (%) (docosahexaenoic acid)	References
Chrysophyceae			
*Pavlova lutheri*	16.2–28.3	3.6–15.5	Mansour et al. [187]
*Pavlova salina*	25.4–28.2	10.2–11.0	Mansour et al. [187]
*Pavlova sp.*	23.5–25.0	8.4–9.2	Dunstan et al. [188]
Bacillariophyceae			
*Amphiprora hyalina*	30	1.9	Dunstan et al. [188]
*P. tricornutum*	9.1–39.0	1.1–5.3	Renaud and Luong-Van [189]
*Biddulphia aurita*	25.6	–	Orcutt and Patterson [190]
*Coscinodiscus sp.*	26	4.6	Orcutt and Patterson [190]
*Nitzschia closterium*	2.6–44.6	01–2.4	Orcutt and Patterson [190]
*Skeletonema costatum*	19.3–26.1	3.9–4.7	Orcutt and Patterson [190]; Molina-Grima et al. [191]
*Thalassiosira stellaris*	25.3	4.8	Salvesen et al. [192]
*Thalassionema nitzschioides*	25.2	1	Dunstan et al. [188]
*Thalassiosira pseudonana*	7.7–32.7	1.4–6.2	Dunstan et al. [188]
Florideophyceae			
*Porphyridium purpureum*	2.9–37.5	–	Matos et al. [193]
Chlorophyceae			
*Nannochloropsis oculata*	13.0–40.0	0.0–0.6	Zhukova and Titlyanov [194]
Cryptophyceae			
*C. cohnii*	–	25–60	Zhukova and Titlyanov [194]

**Table 2:** Different ingredients for feed formulations with potential use in the aquaculture sector.

Feed ingredients	Protein (%)	Lipid (%)	Carbohydrate (%)	Ash (%)	Gross energy (MJ/kg)	References
Fish meal	66.0	7.8	-	19.4	18.4	Karapanagiotidis et al. [175]
Shrimp meal	68.6	3.9	-	16.3	-	Catacutan [173]
Meat and bone meal	46.8	9.6	-	34.1	-	Catacutan [173]
Soybean meal	44.0	2.2	39.0	6.1	18.2	Shields and Lupatsch [195]
Wheat meal	12.2	2.9	69.0	1.6	16.8	Shields and Lupatsch [195]
Brewer yeast	49.6	4.6	31.6	8.0	-	Onofre et al. [177]
*Tenebrio molitor*	61.0	28.6	-	4.1	26.9	Mastoraki et al. [196]
*Musca domestica*	58.5	23.1	-	7.4	24.8	Mastoraki et al. [176]
*Chlorella vulgaris*	55.8	11.2	-	5.2	22.0	Karapanagiotidis et al. [175]
*Schizochytrium*	12.5	40.2	38.9	8.4	25.6	Schields and Lupatsch [195]
*Arthrospira platensis*	55.8	14.2	22.2	7.8	22.7	Tibbetts [66]
*Microchloropsis gaditana*	48.5	21.6	-	8.6	22.7	Karapanagiotidis et al. [175]

**Table 3:** Microalgae inclusion in different aquaculture species.

Aquaculture species	Microalgae species	Inclusion level (up to) %	Results compared to control	References
Nile tilapia	Defatted *Nannochloropsis oculata* (replacing fish meal)	8	Weight gain of 504.3% and lipid retention increase of 1%.	Sarker et al. [179]
	*Schizochytrium sp*. (replacing fish oil)	6.2	Microalgae did not affect protein retention and survival.	
			Increase of DHA about 3.6%.	
Gilthead seabream	*Chlorella vulgaris*	19	*C. vulgaris* increase significantly lipid retention (20.6%).	Karapanagiotidis et al. [175]
	*Schizochytrium sp*.	11	The 3 microalgae did not affects survival, growth, feed efficiency, and protein retention.	
	*Microchloropsis gaditana*	6.2		
Atlantic salmon (postsmolts)	Defatted and extruded *Nannochloropsis oceanica*	10	Replacement by microalgae did not affect growth, protein, and lipid retention.	Gong et al. [197]
			Lipidic profile similar to control feed.	
Juvenile rainbow trout	*Nannochloropsis sp*. and *Schizochytrium sp*.(replacing fish meal and oil)	7 and 2.5	Increase of total n-6 PUFAs content of 18.5%.	Sarker et al. [181]
	*Nannochloropsis sp* and *Isochrysis sp.* (replacing fish meal and oil)	7 and 2.4	Increase of total n-6 PUFAs content of 19.5%	
European sea bass	Pelletized consortium mainly composed by *Oocystis sp*., *Chlorella stigmatophora, Tetraselmis sp*.	20	Growth parameters were not affected	Pascon et al. [198]
Gibel carp	*Oedocladium sp*.	4	Growth parameters were not affected.	Chen et al. [199]
	*Tribonema sp.*	5	*Tribonema* improve DHA (2%) and EPA content (0.5%).	
Juvenile yellow perch	Defatted *Haematococcus pluvialis*	5	Replacement by microalgae did not affect growth, protein, and lipid retention.	Jiang et al. [200]
Pacific white shrimp	*Schizochytrium sp.* (as meal)	6	DHA content increased by almost 3% but EPA content decreased.	Wang et al. [180]
			Inclusion of microalgae did not affect the survival but affected growth parameters.	
			Possible reason was the imbalance between DHA and EPA	
